# Functional analysis of a dihydroflavonol 4-reductase gene in *Ophiorrhiza japonica* (OjDFR1) reveals its role in the regulation of anthocyanin

**DOI:** 10.7717/peerj.12323

**Published:** 2021-10-20

**Authors:** Wei Sun, Nana Zhou, Cai Feng, Shiyu Sun, Ming Tang, Xiaoxin Tang, Zhigang Ju, Yin Yi

**Affiliations:** 1 Key Laboratory of State Forestry Administration on Biodiversity Conservation in Karst Mountain Area of Southwest of China, School of Life Science, Guizhou Normal University, Guiyang, China; 2 Pharmacy College, Guizhou University of Traditional Chinese Medicine, Guiyang, China; 3 Key Laboratory of Plant Physiology and Development Regulation, School of Life Science, Guizhou Normal University, Guiyang, China

**Keywords:** Anthocyanin biosynthesis, Dihydroflavonol 4-reductase, Biochemical characterization, Flower color, *Ophiorrhiza japonica*

## Abstract

Dihydroflavonol 4-reductase (DFR), a key regulatory enzyme, participated in the biosynthesis of anthocyanins, proanthocyanidins and other flavonoids that essential for plant survival and human health. However, the role of this enzyme in *Ophiorrhiza japonica* is still unknown. Here, three putative *DFR-like* genes were firstly isolated from *O. japonica*. Phylogenetic analysis indicated that *OjDFR1* was classified into DFR subgroup, while the rest two were clustered into other NADPH-dependent reductases. Then, functions of the three genes were further characterized. Expression analysis showed that *OjDFR1* transcripts had strong correlations with the accumulation pattern of anthocyanin during the flower developmental, whereas other two were not, this suggested the potential roles of *OjDFR1* in anthocyanin biosynthesis. Subsequently, all three clones were functionally expressed in *Escherichia coli*, but confirming that only *OjDFR1* encode active DFR proteins that catalyzed the reduction of dihydroflavonols to leucoanthocyanidin. Consistant with the biochemical assay results, overexpressing *OjDFR1* in Arabidopsis *tt3-1* mutant successfully restored the deficiency of anthocyanin and proanthocyanidin, hinting its function as DFR in planta. Additionally, heterologous expression of *OjDFR1* in transgenic tobacco contributed to darker flower color *via* up-regulating the expressions of endogenous *NtANS* and *NtUFGT*, which suggested that *OjDFR1* was involved in flower color development. In summary, this study validates the functions of *OjDFR1* and expands our understanding of anthocyanin biosynthesis in *O. japonica*.

## Introduction

Anthocyanins, a kind of flavonoids, were first used to denote the blue pigments of flowers in 1835 (Shibata, Shibata & Kasiwagi, 1919). They are water-soluble and occur widely in the plant kingdom. Anthocyanins play a significant role during plant diverse development processes. Firstly, they are vital coloring pigments that impart wide range colors (pale yellow, orange, pink, red, purple, and blue) to different organs of plants. Simultaneously, these bright colors let plants more outstanding, thus attracting pollinators and seed dispersers for the purpose of breeding (Sara, Pierdomenico & Silvia, 2020; Tanaka, Sasaki & Ohmiya, 2008; Zhao & Tao, 2015). In addition, anthocyanins are also produced for protecting plants from various biotic and abiotic stresses such as fungal infection, insect attacks, high/low temperatures, sunlight exposure, drought, salty soils, as well as heavy metals (Ferreyra, Rius & Casati, 2012). During the last decade, great many researches have proved that anthocyanins are beneficial not only for plants, but also for human health because of their antioxidant, anti-viral, anti-microbial, anti-inflammatory and anticancer actions (Sonia, Diego & Cristina, 2010; Dharmawansa, David & Vasantha, 2020).

**Figure 1:**
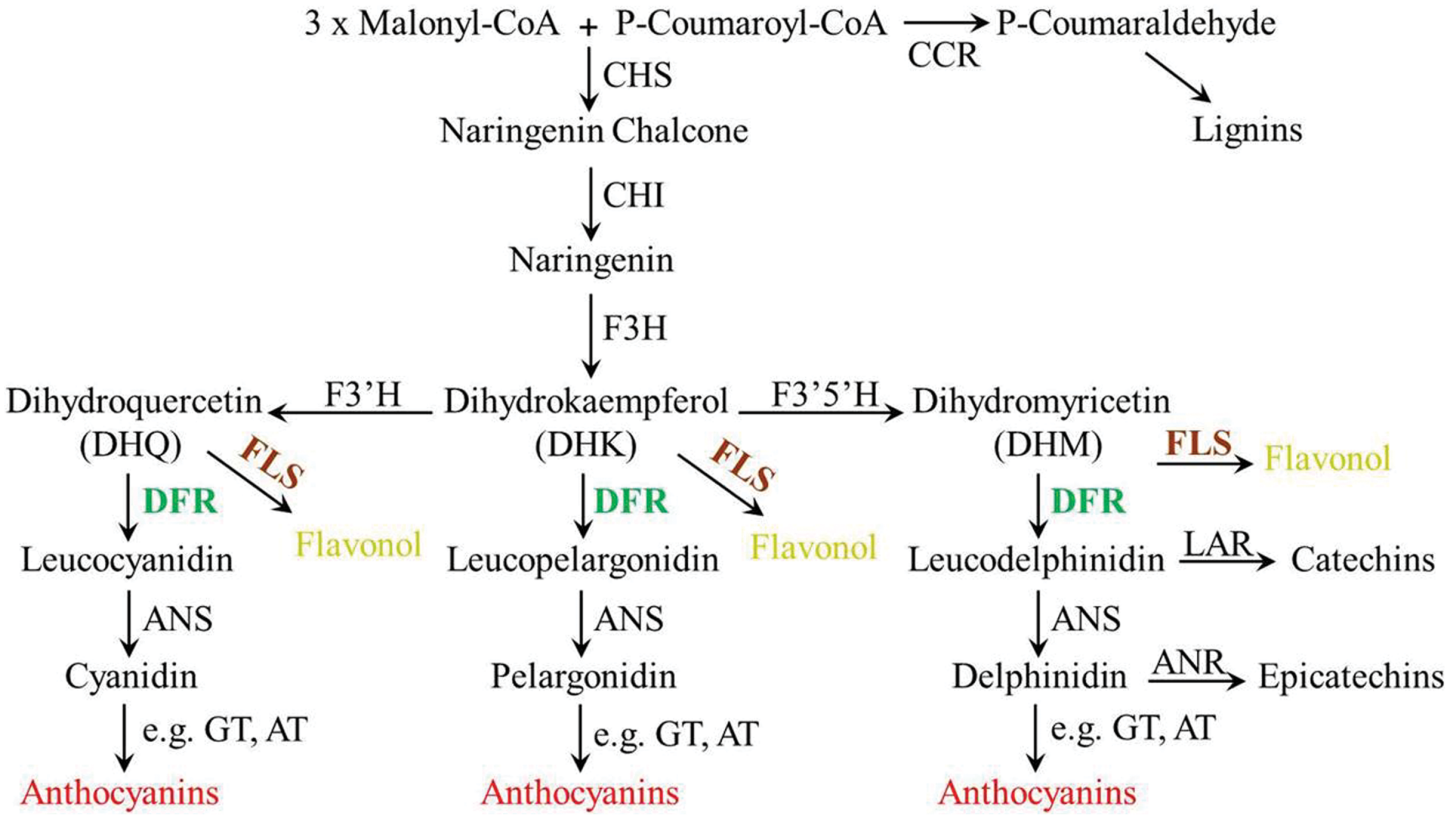
Diagrammatic representation of the lignins, flavonols, proanthocyanidins and anthocyanins biosynthetic pathways in plants. CHS, chalcone synthase; CCR, cinnamoyl CoA reductase; CHI, chalcone isomerase; F3H, flavanone 3-hydroxylase; F3′H, flavonoid 3′-hydroxylase; F3′5′H, flavonoid 3′5′-hydroxylase; FLS, flavonol synthase; DFR, dihydroflavonol 4-reductase; LAR, leucoanthocyanidin reductase; ANS, anthocyanidin synthase; ANR, anthocyanidin reductase; UFGT, anthocyanidin 3-*O*-glucosyltransferase; AT, anthocyanin aromatic acyltransferase.

Anthocyanin biosynthesis involves many enzymes, among them, dihydroflavonol 4-reductase (DFR) is a rate-limited enzyme that controls the carbon flux direction of anthocyanin pathway, thereby leading to strikingly different anthocyanin profiles (Xie et al., 2004; Miyagawa et al., 2015). DFR catalyzes the conversion of three colorless dihydroflavonols (DHM, DHK, DHQ) to the corresponding leucoanthocyanidin and is recognized as a pivotal regulatory point during the biosynthesis of anthocyanins (Fig. 1) (Ni et al., 2020). In general, DFR can be classified into three types because of the difference at amino acid residue 134 (Johnson et al., 2001; Xie et al., 2018). First, there are the asparagine-type (Asn-type) DFRs, which have an Asn at position 134 and are diffusely distributed in plants. Second, there are the aspartic acid-type (Asp-type) DFRs, which contain an Asp at this position and cannot reduce DHK efficiently. And the third type is called non-Asn/Asp-type DFRs, which possess neither Asn nor Asp. Meanwhlie, previous studies have also reported that DFR is commonly found as a single gene in several plants, such as *Arabidopsis thaliana*, *Lycopersicon esculentum*, *Vitis vinifera*, *Antirrhinum majus* and *Oryza sativa* (Shirley, Hanley & Goodman, 1992; Bongue-Bartelsman et al., 1994; Sparvoli et al., 1994; Holton & Cornish, 1995; Chen, SanMiguel & Bennetzen, 1998). Although multiple *DFR* genes are existed, only one of them is catalytically active. For example, there are three tandem *DFR* genes in common morning glory (*Ipomoea purpurea*), but only mutation in *DFR-B* will stop the production of anthocyanin (Hoshino, Johzuka-Hisatomi & Iida, 2001). Similarly, multiple *DFR* genes are also found in *Gerbera hybrid*, but only *GDFR1* was expressed in flowers and catalytically active (Helariutta et al., 1993). Additionally, further biochemical analyses reveal that DFR proteins in certain species have substrate specificities, and this can be the main reason for affecting the content and ratios of anthocyanins, thus determine the final colors of plants (Liu et al., 2019).For example, Petunia (*Petunia hybrida*) and *Cymbidium hybrid* are devoid of pelargonidin-type anthocyanins as well as orange-red flowers, the reason is their DFRs cannot efficiently accept monohydroxylated DHK (Meyer et al., 1987; Johnson et al., 1999). So DFR is crucial in anthocyanin pathway, and controlling its expression levels is often regard as most effective for modifying plant colors. The details of DFRs have been extensively studied in many plants (Feng et al., 2021), but very few information is known in *O. japonica*. Our previous analyses showed that pelargonidin-type anthocyanins were not detected in *O. japonica*, therefore, activity study of its DFRs is necessary for interpreting anthocyanin biosynthesis of *O. japonica*.

*Ophiorrhiza japonica*, a precious Chinese medicinal plant, contains many kinds of bioactive compounds and is commonly used to cure ulcers, leprosy, rheumatism and so on (Sun et al., 2019). Anthocyanins as one of effective medicinal constituents in *O. japonica*, information on its metabolism is very limited. Characterization of key enzymes participated in anthocyanin biosynthesis will aid *O. japonica* medicinal properties improvement. In this current study, three putative *DFR*-like genes were isolated from flowers of *O. japonica*, and only one of them was phylogenetically grouped into subclade of DFR, designated as *OjDFR1* and was further functionally characterized both *in vitro* and *in vivo*. Three *DFR*-like genes showed different expression patterns among the organs, and *OjDFR1* transcripts were highly correlated with anthocyanin accumulation. Then we performed enzyme assay with recombinant protein and checked their catalytic activities for different substrates. Meanwhile, complementation assay of *OjDFR1* in Arabidopsis *dfr* (*tt3-1*) mutant displayed that the phenotype of *tt3-1* rosette leaves and seeds was successfully restored. Furthermore, transgenic tobacco plants overexpressing *OjDFR1* revealed that *OjDFR1* could interact with tobacco anthocyanin pathway enzymes *in vivo* to produce darker flower color. Totally, the above results highlight the importance of *OjDFR1* in regulating anthocyanin biosynthesis and bring a better understanding of the *O. japonica* anthocyanin biosynthetic pathway.

## Materials & methods

### Plant materials

*O. japonica* materials used in this study were grown on the mountain in Shibing, Guizhou Province. For gene isolation and tissue-specific expression analysis, different samples including flowers of four developmental stages (1–4) and six vegetative tissues, *i.e*., flowers (Fl), roots (Ro), stems (St), leaves (Le), scapes (Sc) and calyxes (Ca) were collected as described in our previous paper (Sun et al., 2019). Arabidopsis mutant (*tt3-1*, AT5G42800, ABRC stock number: CS84) was in Landsberg-0 (Ler) ecotypic background and grown at 22 °C with a 16 h light/8 h dark photoperiod. For anthocyanin measurement and RT-PCR analysis, Arabidopsis seedlings of wild type, mutant and transgenic plants cultivated on 1/2 MS medium containing 3% sucrose were obtained. Tobacco plants (Wild-type and transgenic plants) were grown in the greenhouse, the flowers at full-bloom stage were harvested and then used for later analysis. All the samples above were frozen without delay by liquid nitrogen and kept at −80 °C.

### Chemical standards

Dihydroquercetin (DHQ), dihydromyricetin (DHM) and dihydrokaempferol (DHK) were bought from Sigma-Aldrich (St. Louis, MO, USA) and prepared as 10 mg/mL solutions in methanol (chromatographic grade). Cyanidin 3-*O*-glucoside for drawing standard curve was also purchased from Sigma-Aldrich and diluted as one mg/mL solutions in methanol (St. Louis, MO, USA).

### Gene cloning and sequence analysis

Flowers of *O. japonica* were selected for RNA extraction by using RNA pure Plant Kit (CWBIO, China) according to the instructions. Subsequently, one μg total RNA was used to synthesize the cDNA through the method described previously (Sun et al., 2019). Specific primers for cloning the full-length gene coding sequence were designed based on the assembled transcriptomic information. After PCR, the products of appropriate length were sub-cloned into the pMD18-T vector (Takara, Japan) and verified by sequencing. The list of primers used for gene cloning is provided in Table S1. Multisequence alignment of deduced protein sequences was analyzed using DNAMAN 6.0. And the Neighbor-Joining phylogenetic tree was constructed with MEGA version 7.0 software using 1,000 bootstrap replicates.

### Quantitative real-time PCR analysis

RNA extraction and cDNA synthesis of flowers and other vegetative tissues were performed using the methods above. Primers for real-time amplification were designed by IDT1 and shown in Table S1. Then, qRT-PCR reactions were carried out with the BioRad CFX96 Real-Time PCR System (BIO-RAD, Hercules, CA, USA) and TransStart^®^ Green qPCR SuperMix (TRANSGEN, China). Thermal cycling conditions were 95 °C for 60 s, followed by 40 cycles of 95 °C for 5 s and 60 °C for 60 s. The *OjActin* gene and *NtTubA1* gene were chosen as the internal reference for *O. japonica* and tabacum samples respectively. Each experiment sample was conducted in triplicate, and the relative transcript levels of target genes were analyzed by 2^−∆∆Ct^ method. Meanwhile, melting curve analysis and agarose gel electrophoresis were also employed for confirming the purity of PCR products.

### Plasmid construction

In order to obtain His-tagged fusions of OjDFR1, its coding sequence containing the *EcoR* I and *Hind* III restriction enzymes sites was cloned into the pET-32a expression vector. Simultaneously, the complete ORF of *OjDFR1* was also inserted into the binary vector pBI121 that previously digested with *Xba* I and *BamH* I. Then above resulting plasmids were transformed into competent cells and verified by sequencing. Primers used to generate the recombinant are present in Table S1.

### Production of recombinant OjDFR1 protein and *in vitro* enzyme assay

The recombinant construct pET-32a-OjDFR1 and empty vector were introduced into *E. coli* strain BL21 (DE3) by the heat shock method. Next day, a single colony containing expression plasmid was inoculated in Luria–Bertani (LB) medium and grown at 37 °C with shaking 200 rpm until OD_600_ reached 0.6. For induction, 0.2 mm of isopropyl-β-d-thiogalactopyranoside (IPTG) was added and the cells were further incubated at 15 °C for 24 h. After that, the cells were harvested centrifugation at 5,000 rpm for 10 min at 4° and resuspended in phosphate-buffered saline (PBS) for sonication, then the debris was removed by centrifugation at 12,000 rpm for 5 min at 4°. Subsequently, the His-tagged OjDFR1 proteins were purified by Ni-NTA pre-packed column (TransGen, China) and its purity was examined by using sodium dodecyl sulfate polyacrylamide gel electrophoresis (SDS-PAGE). Protein concentration was estimated by NanoDrop 1,000 (Thermo scientific, Waltham, MA, USA) Spectrophotometer before i*n vitro* enzymatic assays.

Three dihydroflavonols (DHQ, DHM and DHK) were selected as experimental substrates to analyze the substrate specificities of OjDFR1 following the methods depicted previously (Cheng et al., 2013). Shortly, a 500 µL reaction mixture containing 100 mm TrisHCl buffer (pH 7.0), 20 mm NADPH (50 µL), 10 mg/mL substrate (10 µL) and 35 µg OjDFR1 enzyme extract was conducted at 30 °C for 30 min. And the reaction products were identified by HPLC using a Shimadzu HPLC system with detection at 280 nm. An ACCHROM XUnion C18 column was used with the temperature at 30 °C and 20 µL samples were eluted with 1% H_3_PO_4_ (solvent A) and methanol (solvent B) according to the program as follows: 0 min, 15% B; 0–20 min, 15–60% B; 20–30 min, 60–15% B at flow rate of one mL/min.

### Transformation of OjDFR1 into *A. thaliana* and tobacco

To further characterize the function of *OjDFR1*, the resulting vector pBI121-OjDFR1 and empty expression vector were maintained in *Agrobacterium tumefaciens* strain GV3101 by freeze-thaw method. Next, about 5∼6-week old *tt3-1* mutant plants were used for genetic transformation followed the standard floral dipping transformation procedure (Clough & Bent, 1998). Transformants were screened on 1/2 MS medium with 50 mg L^−1^ kanamycin to obtain T2 seeds. T2 transgenic seeds as well as wild type and mutant seeds were then cultured on anthocyanin induction media (1/2 MS medium supplemented with 3% sucrose) to set seedlings for phenotypic investigations and metabolite analysis. Meanwhile, the construct pBI121-OjDFR1 was also transformed into tobacco *via* the method described in previous reports (Imogen et al., 2006). The transgenic seedlings of tobacco (T1) were grown in green house, and their flower color was observed after flowering. For detecting the expression levels of *OjDFR1*, Arabidopsis *actin-1* and *NtTubA1* were selected as internal control genes.

### Total anthocyanin quantifications by HPLC

The contents of anthocyanin were measured in both Arabidopsis seedlings and tobacco flowers according to previous method reported by predecessors (Li et al., 2017). Briefly, 0.1 g samples of seedlings as well as tobacco flowers were mashed into a fine powder and placed into extraction solution (water: methanol: hydrochloric acid = 75:24:1) at 4° C for 12 h. After centrifugation at 12,000 rpm for 5 min at 4° C, the supernatant was collected and filtered by a 0.22 μm nylon membrane filter for the following analysis. Chromatographic detection was performed on a Shimadzu HPLC system with the absorbance at 520 nm based on the procedure described by Li et al. (2017). Total anthocyanin content was quantified by using the external standard curve calibration of cyanidin 3-*O*-glucoside with three biological replicates (Fanali et al., 2001).

### Statistical analysis

All experiments were repeated at least three times. The data were expressed as mean ± SD (standard deviation). We used a *t*-test to test for significant differences in the anthocyanin contents as well as gene expressions between wild type and transgenic tobaccos (*OjDFR1*-4 and *OjDFR1*-5). Difference with *P* < 0.05 was considered statistically significant.

## Results

### Isolation and phylogenetic analysis of *DFR*-like genes from *Ophiorrhiza japonica*

After *in situ* TBLASTN search of *O. japonica* transcriptomic database, three putative genes which encoded NADP-binding reductases were obtained. Among them, one gene was named as *OjDFR1* (cDNA sequences listed in the Supplementary Materials), which was most likely to be the *bona fide DFR*, because its protein sharing 69.74% identities to petunia DFR, and 67.98% identities to *Nicotiana tabacum* DFR. On the contrary, other two genes might encode ANR-like and FR proteins on the basis of manual BLASTX search, and thus were tentatively designated as *OjANR1* and *OjFR1*.

**Figure 2:**
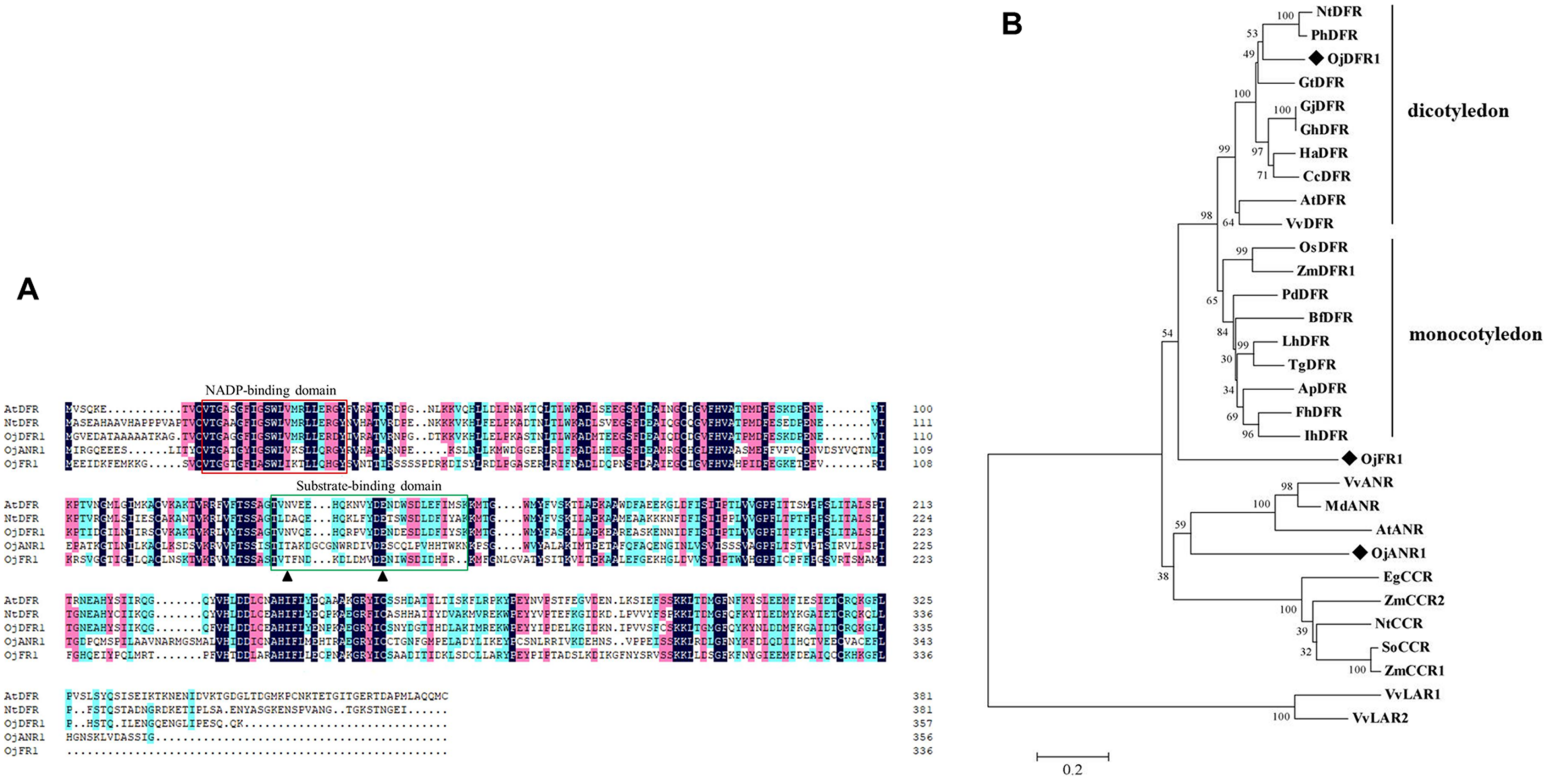
Amino acid sequences alignment and phylogenetic analysis of DFR-like proteins in *O. japonica* with proteins from other species. (A) Multiple sequence alignment of *O. japonica* proteins with DFR from other species. The putative NADP-binding site and presumed substrate-binding region were marked by red and green boxes. Black triangles indicated the 134rd and 145rd amino acid residue, which is important for substrate specificity of DFR. (B) Phylogenetic analysis of NADPH-dependent reductase proteins from *O. japonica* and other plant species. Proteins of *O. japonica* were indicated with black rhombus. GenBank accession numbers are as follows: *Agapanthus praecox* ApDFR (BAE78769), *Bromheadia finlaysoniana* BfDFR (AAB62873.1), *Freesia hybrid* FhDFR (KU132393), *Iris hollandica* IhDFR (BAF93856.1), *Lilium hybrida* LhDFR (BAB40789.1), *Oryza sativa* OsDFR (BAA36183.1), *Phoenix dactylifera* PdDFR (XP_008797532.1), *Tulipa gesneriana* TgDFR (BAH98155.1), *Zea mays* ZmDFR1 (NP_001152467.2), *Gentiana triflora* GtDFR (BAA12736.1), *Arabidopsis thaliana* AtDFR (BAD95233.1), *Nicotiana tabacum* NtDFR (NP_001312559.1), *Gerbera jamesonii* GjDFR (KF734593), *Gerbera hybrida* GhDFR (AKN56969.1), *Petunia hybrida* PhDFR (CAA56160), *Vitis vinifera* VvDFR (X75964), *Helianthus annuus* HaDFR (XP_022001438.1), *Callistephus chinensis* CcDFR (CAA91922.1), *Arabidopsis thaliana* AtANR (Q9SEV0.2), *Vitis vinifera* VvLAR1 (AAZ82410), VvLAR2 (AAZ82411), VvANR (BAD89742), *Malus domestica* MdANR (AEL79861.1), *Nicotiana tabacum* NtCCR (A47101), *Saccharum officinarum* SoCCR (AJ231134), *Zea mays* ZmCCR1 (Y13734), ZmCCR2 (Y15069) and *Eucalyptus gunnii* EgCCR (X97433).

Multisequence alignment with AtDFR and NtDFR showed that the three *O. japonica* proteins had the highly conserved NADP-binding motif and the substrate-binding domain at their N terminus (Fig. 2A). And the amino acid residue (at position 134) which is important for substrate specificity of DFR is Asn in OjDFR1 (encoding 357 amino acid residues with a calculated molecular mass of 39.87 kD) indicating that OjDFR1 may belong to the Asn-type DFR and catalyze the reduction of three dihydroflavonols (DHK, DHQ, and DHM) to leucoanthocyanidins. Moreover, phylogenetic analysis between OjFRs and other NADPH-dependent reductases (DFR, LAR, ANR, and CCR) are consistent with the above analyses. As shown in Fig. 2B, OjDFR1 protein was grouped into the eudicots subclade of DFR and exhibited most similar to the DFR from petunia. While, other two genes from *O. japonica* clustered outside the *DFR* branch, one fell into the subgroup of ANR, and another did not belong to a clear subclade, suggesting they might participate in other NADPH-dependent reduction during flavonoid biosynthesis.

### Expression analysis of *DFR*-like genes in different organs

**Figure 3:**
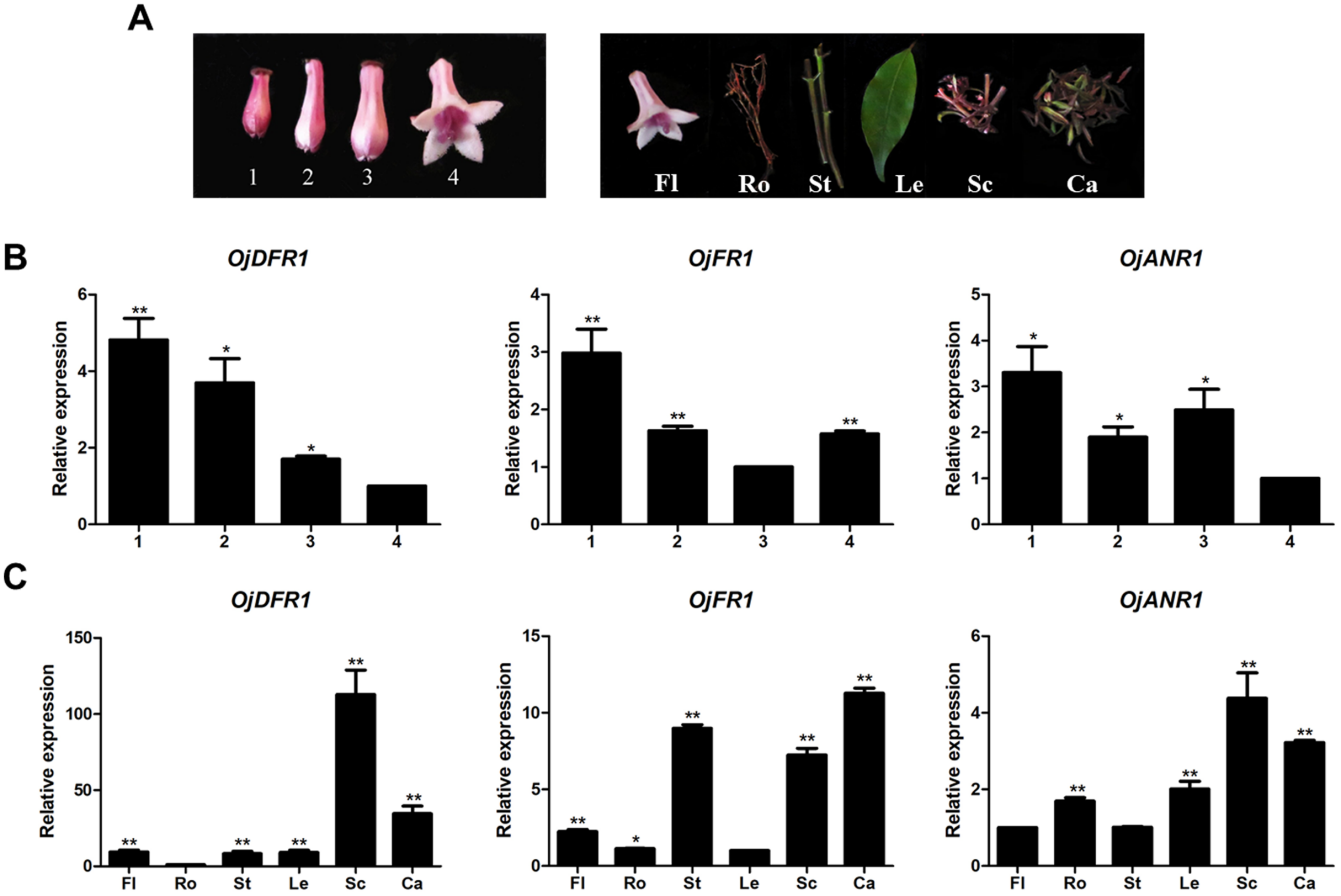
Expression profiles of DFR-like genes in *O. japonica*. (A) Four developmental stages of flowers and different tissues. (B) Relative expression levels of *OjDFR-like* genes at four flower developmental stages. (C) Relative expression levels of *OjDFR-like* genes in different tissues. Results represent means ± SD from three biological replicates. Asterisks above the bars indicate significant difference between the samples judged by Student’s *t*-test (*P < 0.05, **P < 0.01).

Before further analysis, all three genes were subjected to analyze their expressions at four flower developmental stages (1–4) and six different organs (Fig. 3A). It was observed that transcripts of three genes all exhibited significant spatial and temporal specificity. The relative levels of *OjDFR1* transcripts peaked at stage 1 and decreased gradually with the flower development. For *OjFR1* and *OjANR1* genes, although they were also highly expressed in stage 1, but their transcript levels did not show correlation with the accumulation pattern of anthocyanin (Fig. 3B). Furthermore, expression pattern analyses in various tissues displayed that *OjDFR1* showed higher transcript levels in scapes and calyxs accumulating high levels of anthocyanin than that of *OjFR1* and *OjANR1* (Fig. 3C). These data suggest that *OjDFR1* appears to fulfill crucial roles in the anthocyanin biosynthesis in *O. japonica*.

### Heterologous expression in *Escherichia coli* and *in vitro* biochemical characterization

**Figure 4:**
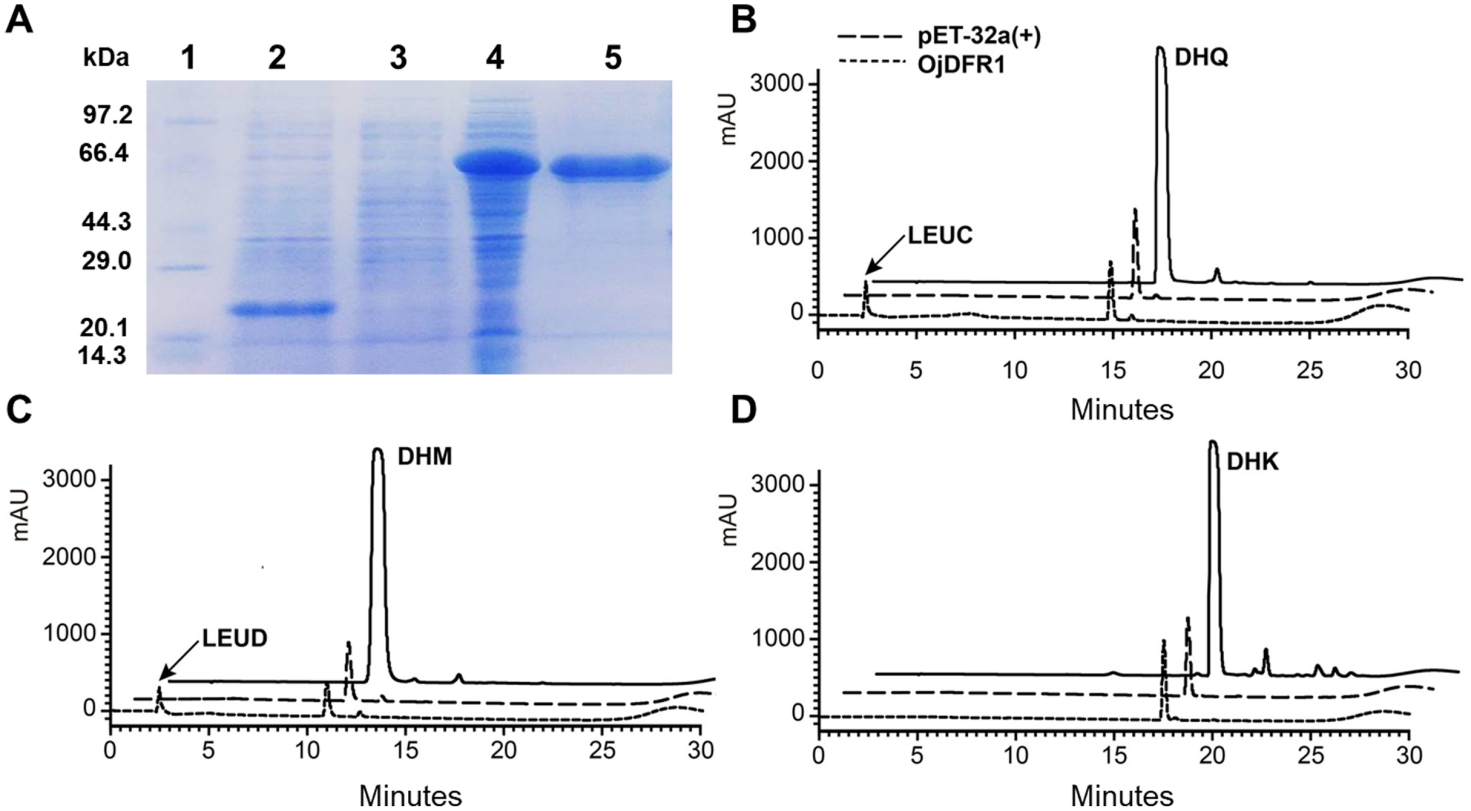
Biochemical assays of recombinant OjDFR1. (A) Expression of *OjDFR1* in *E. coli*. (1) Maker (2) Total soluble protein from *E. coli* expressing pET-32a (+) vector (3) Total soluble protein from *E. coli* expressing *OjDFR1* prior to induction by IPTG (4) A total of 24 h after induction (5) Purified OjDFR1. Enzymatic reaction contained DHQ (B), DHM (C), and DHK (D) as substrates, NADPH as well as protein extracts from *E. coli*. harboring pET-32a and OjDFR1. LEUC and LEUD represent leucocyanindin and leucodelphinidin respectively.

To provide direct evidence that the *OjDFR1* encoded the DFR enzyme, *in vitro* enzyme activity assay was implemented. The cDNA of *OjDFR1* was therefore subcloned into the pET-32a expression vector and soluble recombinant proteins were successfully purified followed by SDS-PAGE identification (Fig. 4A). Next, the purified recombinant proteins were incubated with DHQ, DHM and DHK in the presence of NADPH, and the respective reaction products were characterized by HPLC through comparing to the UV spectra and the relative retention time (Cheng et al., 2013). As shown in Fig. 4B and 4C, HPLC analyses detected at 280 nm revealed that new peak was observed from reactions using DHQ and DHM as substrate, and these two peaks were not observed from control (protein from *E. coli* carrying the pET-32a empty vector). However, reduction of DHK to the respective leucopelargonidin was not observed (Fig. 4D). In fact, the recombinant OjFR1 and OjANR1 were also tested by DHQ, DHM and DHK, but no formation of the respective leucoanthocyanidin was observed (Fig. S1).

### Functional analysis of *OjDFR1* in Arabidopsis *tt3-1* mutants

**Figure 5:**
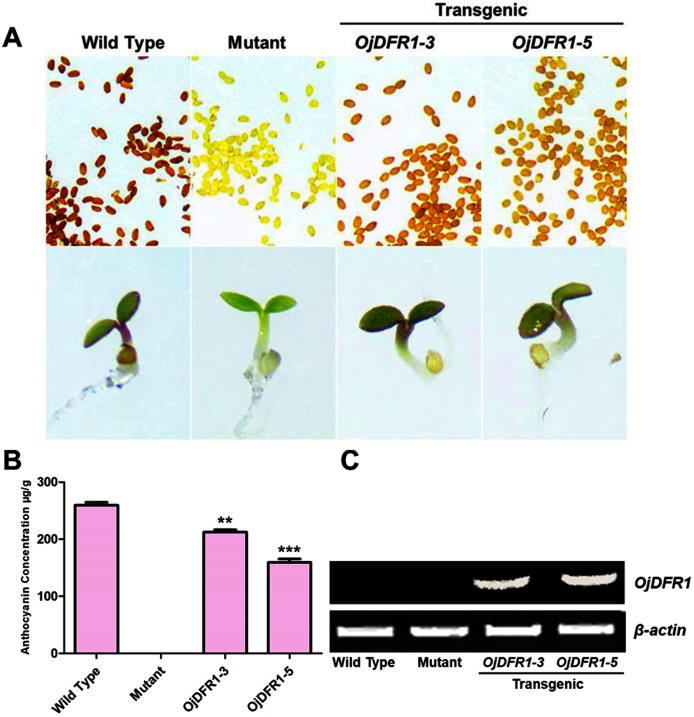
Complementation of DFR function in Arabidopsis *tt3-1* mutant. (A) Phenotype of the wild type (Ler), mutant (*tt3-1*) and T2 transgenic lines. (B) Total contents of anthocyanins in Arabidopsis seedlings. (C) Expressional analysis of *OjDFR1* in wild-type, mutant and transgenic lines. Results correspond to means from three biological replicates. Asterisks indicate significant differences between means of wild-type and transgenic plants calculated by Student’s *t*-test (**P < 0.01, ***P < 0.001).

To validate the functional identity of *OjDFR1*, its cDNA was subcloned into the pBI121 expression vector containing the CaMV 35S promoter. *Via* floral dip method, *OjDFR1* was introduced into Arabidopsis *tt3-1*mutant which failed to accumulate brown proanthocyanidins in the seeds and anthocyanins in the cotyledons and hypocotyls. Transgenic plants were selected on MS medium with 50 mg/L kanamycin, and then seeds of wild-type, *tt3-1* as well as T2 transgenic lines were grown for 7 days on medium supplemented with 3% sucrose. Phenotypic investigation showed that transgenic *OjDFR1* plants successfully restored the coloration of their seeds and hypocotyls (Fig. 5A), while the empty vector control was still green (raw data). In order to confirm this phenotype was produced by ectopic expression of *OjDFR1*, RT-PCR was carried out. As expected, *OjDFR1* was highly expressed in transgenic plants, and no amplicons were observed in the control (Fig. 5C).

**Figure 6:**
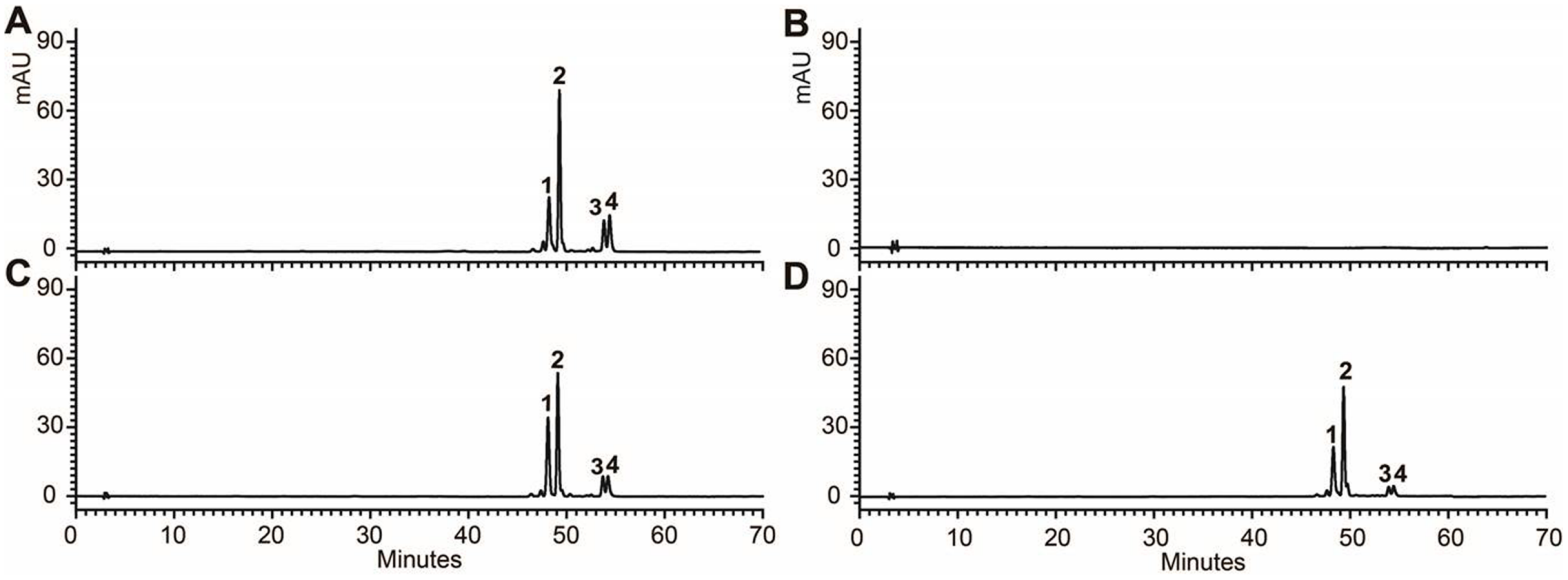
FHPLC analyses of anthocyanins in Arabidopsis seedlings. HPLC chromatograms of the samples from seedlings of wild-type (A), mutant (B) and transgenic lines (C, D).

In addition, 7-day-old seedlings were also used to quantitatively determine the amounts of anthocyanins, and the results indicated that anthocyanins level in transgenic Arabidopsis was higher than mutant, and similar to wild type (Fig. 5B). As results present in Fig. 6, anthocyanins were not detected in *tt3-1*, but these absent peaks were fully complemented in transgenic seedlings expressing *OjDFR1*, though the contents were lower. Totally, our anthocyanin analyses strongly prove that *O. japonica DFR* gene is functional for the biosynthesis of proanthocyanidins and anthocyanins *in vivo*.

### *OjDFR1* contributes to darker flower color in transgenic tobacco

**Figure 7:**
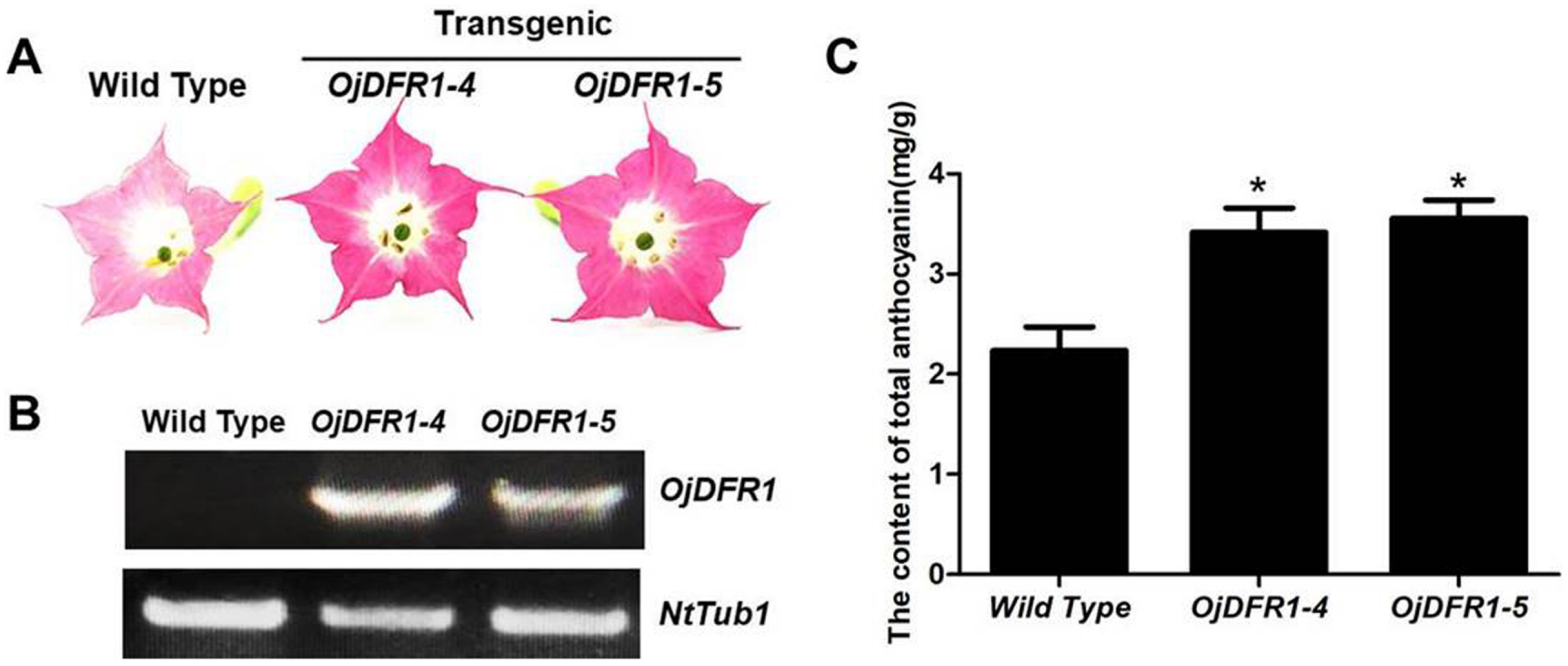
Effect of *OjDFR1* on anthocyanin accumulation in transgenic tobacco flowers. (A) Tobacco flowers of wild-type and transgenic lines. (B) Expression profiles of *OjDFR1* in flowers of transgenic tobacco. (C) Quantitation of anthocyanin accumulation levels in transgenic tobacco flowers with HPLC. Results correspond to means from three biological replicates. Asterisks indicate significant differences between means of wild-type and transgenic plants calculated by Student’s *t*-test (*P < 0.05).

**Figure 8:**
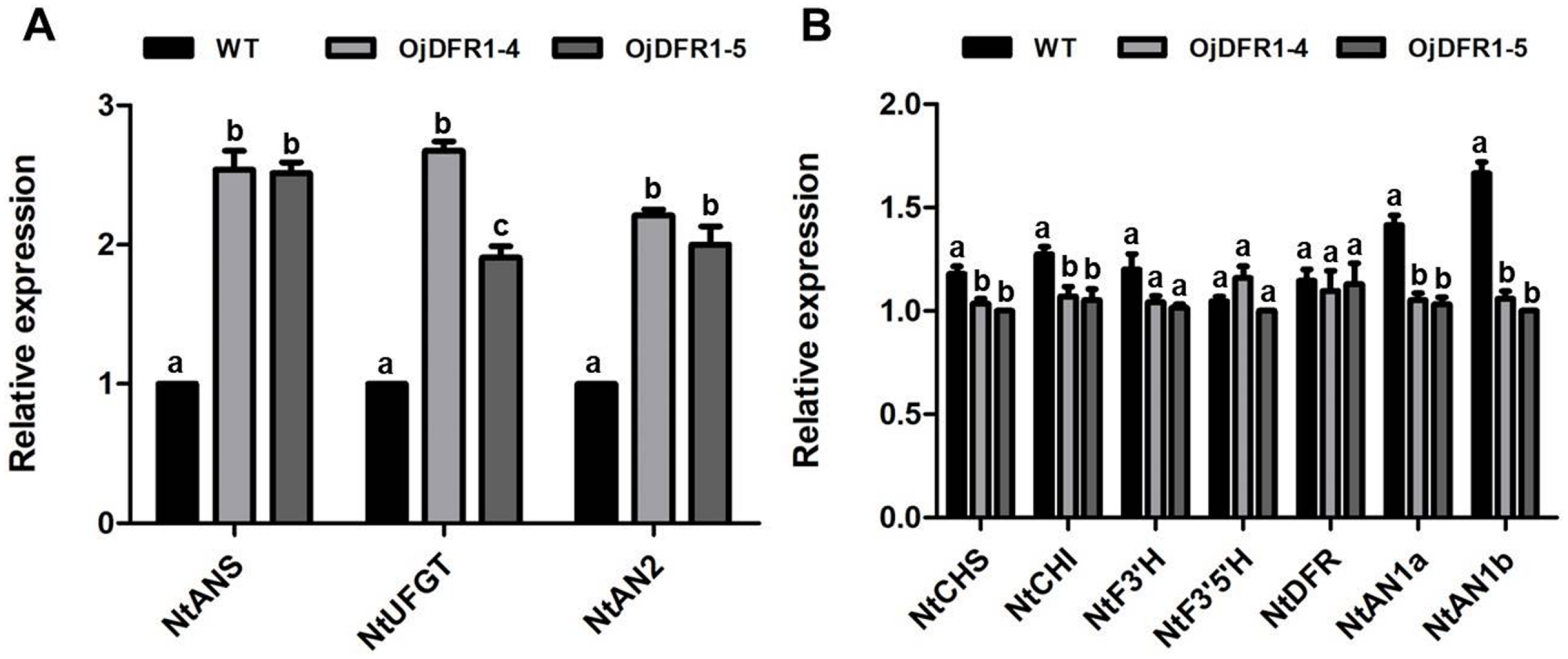
Expression analysis of endogenous anthocyanin biosynthetic genes in corollas of transgenic tobacco. (A) Expression profiles of structure genes in corollas of transgenic tobacco. (B) Expression profiles of regulatory genes in corollas of transgenic tobacco. Results represent means ± SD from three biological replicates. Different letters above the bars indicate significant difference between the samples judged by Student’s *t*-test (*P* < 0.01).

In order to characterize the effects of *OjDFR1* transgene on anthocyanin profiles in flowers, it was overexpressed in tobacco plants. Totally 12 independent transgenic lines that confirmed by gene-specific PCR analysis were obtained. Based on visual observations, transgenic tobacco plants possessed darker pink flowers than wild type (Fig. 7A). Then the presence of *OjDFR1* was examined by RT-PCR (Fig. 7B). Additionally, anthocyanin levels in corollas were also measured through HPLC (Fig. S2). Compared with control, corollas of transgenic tobacoo overexpressing *OjDFR1* contained significantly higher amount of anthocyanins on fresh weight basis (Fig. 7C), which implying OjDFR1 protein might interact with the endogenous enzymes of anthocyanin pathways in tobacco and lead to increased anthocyanin accumulation in transgenic flowers. For further investigate the effect of *OjDFR1* on endogenous tobacco in anthocyanin biosynthetic genes, we performed qPCR analysis. Among these genes (*NtCHS*, *NtCHI*, *NtF3’H*, *NtF3’5’H*, *NtDFR*, *NtANS*, *NtUFGT*, *NtAN1a*, *NtAN1b* and *NtAN2*), the expressions of *NtANS*, *NtUFGT* and *NtAN2* were higher in transgenic tobacco flowers, whereas the transcripts levels of *NtCHS*, *NtCHI*, *NtAN1a* and *NtAN1b* were slightly lower in transgenic flowers than in wild-type (Fig. 8).

## Discussion

DFR, one of the members of short chain dehydrogenase/reductase (SDR) superfamily, fulfills important regulatory role in the formation of anthocyanins and controls the carbon flux into different anthocyanin biosynthetic branches which results in various anthocyanin profiles (Li et al., 2017). Although single or multiple *DFR* genes are characterized in many plant species, no detailed identification of *DFR* in *O. japonica* has been performed (Chen et al., 2020). In this study, three NADPH-dependent reductase genes were firstly cloned from *O. japonica*. Among them, OjDFR1 tended to be DFR-like proteins, while other two proteins were likely to be ANR and FR respectively according to BLASTX search. Amino acid alignment showed that all these three proteins had the typical NADPH-binding motif and the substrate-binding domain. Correspondingly, phylogenic tree of NADP-dependent reductases including DFR, ANR, LAR, CCR revealed that OjDFR1 clustered into the subclade of characterized DFRs, which implying the potential “DFR-like” catalytic activity of OjDFR1. As reported previously, many members of protein family have identical or similar functions *in vitro*, and their different roles in plants are largely due to their specific expression patterns (Vieten et al., 2005), so the expression patterns of above three genes were examined temporally and spatially. During flower development, anthocyanin accumulation in *O. japonica* decreased gradually and calyxes were its dominant tissues for the biosynthesis of anthocyanin (Sun et al., 2019). As results present in Fig. 3, the expressions of *OjDFR1* exhibited strong correlations with the accumulation patterns of total anthocyanins in flower development process, but *OjANR* and *OjFR* were not. *OjDFR1* transcript accumulations were similar to *DFR* in *Ginkgo biloba*, Chrysanthemum and *Pyrus communis* which was expressed positively correlated with anthocyanin concentrations (Cheng et al., 2013; Li et al., 2017; Sun et al., 2020). However, in different tissues, relatively higher *OjDFR1* expression was observed in Sc than that in Ca, which is not in line with anthocyanin phenotype. These results suggest that *OjDFR1* is not flower specific and can be involved in the biosynthesis of other flavonoids in *O. japonica* such as proanthocyanin.

Knowledge of DFR biochemical properties is vital for understanding the metabolism of flavonoid, especially its regulation in specific branches. Biochemical studies of DFR have demonstrated that substrate specificity of this enzyme could be determined by the amino acid residue at position 134 (Li et al., 2012). As reported previously, the Asn-type DFRs (contain Asn at posituon 134) could convert all three dihydroflavonols to the respective leucoanthocyanidin (Forkmann & Ruhnau, 1987). However, to our amazement, OjDFR1 belonged to Asn-type DFRs, but no leucopelargonidin (LEUP) was observed in the enzyme activity assay (Fig. 4), which is in accord with the fact that pelargonidin glycosides were not detected in *O. japonica*, and this result is similar to the research of DFR in *Agapanthus praecox ssp.orientalis* and *Freesia hybrid* (Mori et al., 2014; Li et al., 2017; Sun et al., 2019). It is possible that the ability of OjDFR1 catalyzing DHK is lost during the evolution because of the relatively low concentration of DHK in *O. japonica*. Or the residue at position 134 is not an absolute factor to determine the substrate specificity of DFR. Exactly, the DFR from *Grape Hyacinth* which haves a point mutation at amino acid residue 134 was still able to utilize all three dihydroflavonol as substrates (Liu et al., 2019). So these results indicate that the residue at position 134 is not an absolute factor to determine the substrate specificity of DFR. Indeed, domain swapping experiments had illustrated that the ability of DFR for catalyzing dihydrokaempferol was influenced by the first 170 amino acids (Johnson et al., 2001). Taken together, it will be necessary and interesting to carry out site-directed mutagenesis analysis for confirming the contribution of a specialized amino acid residue in the substrate-binding region towards the activity and substrate preference of DFRs.

Functionality of *OjDFR1* was further investigated *via* its introduction in Arabidopsis *tt3-1* mutants, and the results indicated that *OjDFR1* could rescue the pigmentation phenotype of *tt3-1* which demonstrated OjDFR1 as a DFR was functionally active *in vivo* for anthocyanin biosynthesis (Fig. 5A). But results in Fig. 5B revealed that the contents of total anthocyanins in transgenic plants were lower in contrast to wild type, and this can be speculated that the catalytic efficiency of OjDFR1 might be weaker than the DFR of Arabidopsis. As known, wild-type Arabidopsis accumulate pelargonidin in the seedlings (Sun et al., 2016) which corresponds to peak 3 in our HPLC experiments (Fig. 6). So, apparently, the successful restoration of peak 3 in transgenic Arabidopsis was not consistent to the results of OjDFR1 biochemical properties assays. On one hand, this contradiction might be ascribed to the very low catalytic efficiency of OjDFR1 towards DHK which was lost in the course of preparation. On the other hand, the enzymes participated in the biosynthesis of natural product are promiscuous, post-translational modification, coenzyme, the relative concentrations of potential substrates and the internal environment of plants are all the influenced factors to determine their activity *in vivo*. Overall, OjDFR1 could use DHQ and DHM as substrate in *O. japonica*, DHQ and DHK as substrate in Arabidopsis.

Overexpression was used to evaluate whether or not the OjDFR1 would perform differently in tobacco cells. As shown in Fig. 7A, the transgenic plants overexpressing *OjDFR1* had darker pink flowers, whereas the control produced pale pink flowers, and this coincided well with accumulation levels of anthocyanin (Fig. 7B). Likewise, the significant phenotype of transgenic tobacco mentioned above was also found in the studies of cranberry and *Populus trichocarpa* which hinted the necessary role of DFR for the formation of flower color (Polashock et al., 2002; Huang et al., 2012). In addition, we observed that although the upper of transgenic corolla were darker pink, the lower part was still white as same to the control. Considering that the position of DFR in anthocyanin pathway, we speculate the downstream enzymes (ANS, UFGT or other enzymes) may also play crucial roles in the formation of transgenic tobacco flower color (Ni et al., 2020). Interestingly, overexpression of *OjDFR1* in tobacco resulted in the up-regulation of *NtANS* and *NtUFGT* as well as the regulatory gene *NtAN2* (Fig. 8A). Previously, it was reported that positive feedback regulation of flavonoid biosynthetic genes *via* pathway intermediates was existed in *Arabidopsis thaliana* mutant (Pourcel et al., 2013). Therefore, we proposed that the up-regulatuon of *NtANS* and *NtUFGT* might be due to the positive feedback regulation by anthocyanins accumulation (flavonoid pathway intermediates) resulting from *OjDFR1* overexpression. Meanwhile, it seems that *OjDFR1* overexpression may also affect the regulatory role of *NtAN2*, but whether or not the OjDFR1 can directly interact with R2R3 MYB proteins is not clear, or that the alteration of *NtAN2* expression was due to feed-back from the increased level of intermediates. Out of expectation, HPLC analysis showed that only Cy-type pigments were produced in transgenic plants (Fig. S2). And the deficiency of Dp-type anthocyanins in host tobacco plants is reported for the lack of DHM (Sun et al., 2016). Inconsistent with the results in transgenic *tt3-1*, Pg-type anthocyanins was not detected in transgenic tobacco, and this further demonstrates that the enzymes involved in natural product biosynthesis such as DFR are extraordinarily complicated. Taken together, overexpression of *OjDFR1* gene in tobacco contributes to significant increase of total anthocyanin which is probably attributed to the up-regulation of *NtANS* and *NtUFGT*.

## Conclusions

Collectively, we carried out a comprehensive analysis of *DFR* gene in *O. japonica*. Our results showed that the identified *OjDFR1* gene was associated with color development in flowers. Meanwhile, the OjDFR1 enzyme could utilize DHQ as well as DHM as substrates *in vitro*, and could restore the biosynthesis of anthocyanins and proanthocyanidins in Arabidopsis. Furthermore, the results obtained from transgenic tobacco demonstrated that *OjDFR1* might be a determinant for the categories of anthocyanins aglycons accumulated in *O. japonica*. Therefore, our findings provide a better understanding of anthocyanin biosynthesis in *O. japonica*.

## Supplemental Information

10.7717/peerj.12323/supp-1 Supplemental Information 1 Expression of *OjANR1* and *OjFR1*in *E. coli*.

10.7717/peerj.12323/supp-2 Supplemental Information 2 HPLC analyses of anthocyanins in transgenic tobacco flowers.

10.7717/peerj.12323/supp-3 Supplemental Information 3 List of primers used in this study.

10.7717/peerj.12323/supp-4 Supplemental Information 4 Raw data.

## Additional Information and Declarations

### Data Availability

The following information was supplied regarding data availability: The raw measurements are available in the Supplementary Files.
