# Supplementary figures and images for: Functional analysis of a dihydroflavonol 4-reductase gene in Ophiorrhiza japonica (OjDFR1) reveals its role in the regulation of anthocyanin
Source: PeerJ. 2021 Oct 20;9:e12323. doi: 10.7717/peerj.12323 (PMC8541326; doi:10.7717/peerj.12323)

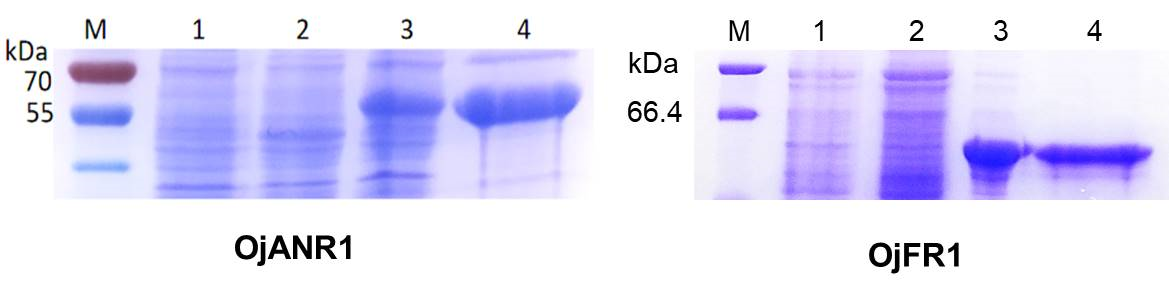

Supplement: Supplemental Information 1 [file peerj-09-12323-s001.png]

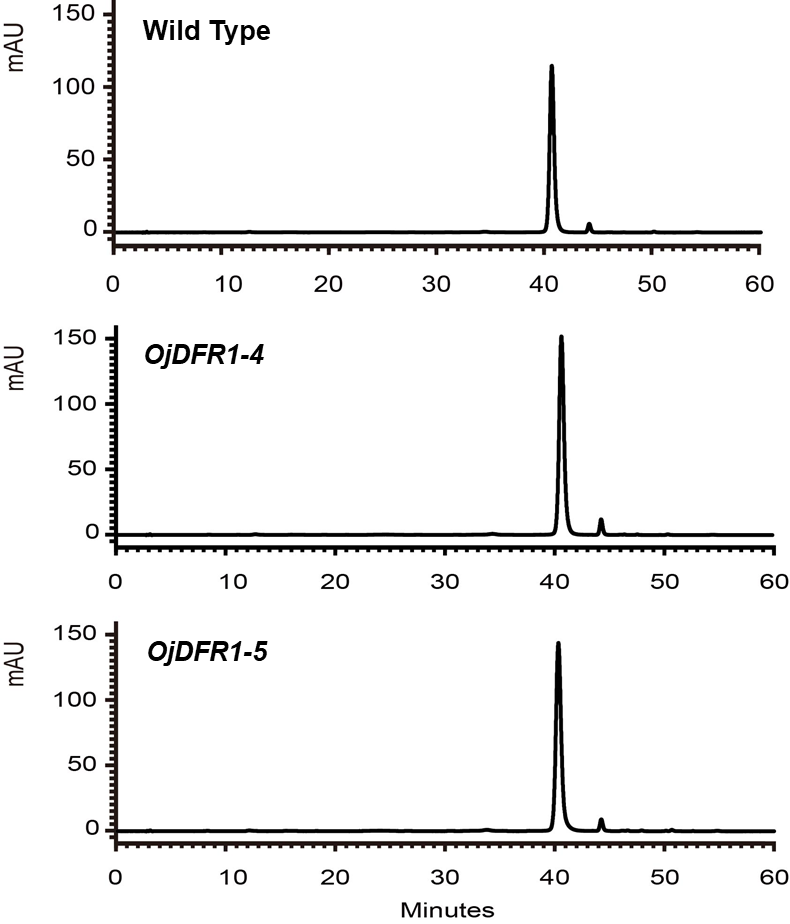

Supplement: Supplemental Information 2 [file peerj-09-12323-s002.png]
