# Supplementary material for: Functional analysis of a dihydroflavonol 4-reductase gene in Ophiorrhiza japonica (OjDFR1) reveals its role in the regulation of anthocyanin
Source: PeerJ. 2021 Oct 20;9:e12323. doi: 10.7717/peerj.12323 (PMC8541326; doi:10.7717/peerj.12323)
Supplement: Supplemental Information 3 [file peerj-09-12323-s003.docx]

**Supplementary Table S1** List of primers used in this study

| **Primer name** | **Primer sequence (5'→3')** |
| --- | --- |
| *OjDFR1-F* | ACGATTCTCACATTCCATCTTC |
| *OjDFR1-R* | CCAGCAAAAATAGGCATCAATG |
| *OjFR1-F* | AATGGAAGAAATAGACAAGT |
| *OjFR1-R* | TGCTTGTCTCGCAGATTAAG |
| *OjANR1-F* | ATCCTCTAGAGATTTAATGG |
| *OjANR1-R* | AATCGTCGACCTGCAGGCAT |
| *OjDFR1-qRT -F* | GCTGAGAAAGAAGCACGAGA |
| *OjDFR1-qRT-R* | GGAATGTTGGCGTGATGAATG |
| *OjFR1-qRT-F* | GATCTGTCGGTGGAACAATAGG |
| *OjFR1-qRT-R* | TGCACTCGAAGTATACACAACTC |
| *OjANR1-qR -F* | GGCAAAGATTATGACTGAGGAAAC |
| *OjANR1-qRT-R* | GGACCAGCAACAGAAGAAGATA |
| *NtCHS-qRT-F* | TGACACCCACTTGGATAGTTTAG |
| *NtCHS-qRT-R* | CGACCTCTGGAATTGGATCAG |
| *NtCHI-qRT-F* | CTTTTCTCGCCGCTAAATG |
| *NtCHI-qRT-R* | TTTCTGCCACCTTCTCTG |
| *NtF3'H-qRT-F* | AGGCTCAACACTTCTCGT |
| *NtF3'H-qRT-R* | CATCAACTTTGGGCTTCT |
| *NtF3'5'H-qRT-F* | CGCACTACCATACTTAGGAGCCAT |
| *NtF3'5'H-qRT-R* | CAGCATCAGGAGTAGAAGCAACAG |
| *NtDFR-qRT-F* | AACCAACAGTCAGGGGAATG |
| *NtDFR-qRT-R* | TTGGACATCGACAGTTCCAG |
| *NtANS-qRT-F* | TGGCGTTGAAGCTCATACTG |
| *NtANS-qRT-R* | GGAATTAGGCACACACTTTGC |
| *NtUFGT-qRT-F* | GAGTGCATTGGATGCCTTTT |
| *NtUFGT-qRT-R* | CCAGCTCCATTAGGTCCTTG |
| *NtTubA1-qRT-F* | CTCCTATGCTCCTGTCATTTC |
| *NtTubA1-qRT-R* | GGCGAGGATCACACTTAAC |
| *NtAN2-qRT-F* | GAAGAAAGGTGCATGGACTG |
| *NtAN2-qRT-R* | TCTGCAGCTCTTTCTGCATC |
| *NtAN1a- qRT-F* | ACCATTCTCGAACACCGAAG |
| *NtAN1a- qRT-R* | TGCTAGGGCACAATGTGAAG |
| *NtAN1b- qRT-F* | CTTGAACACTTCTCAAACCGA |
| *NtAN1b- qRT-R* | TGCTAGGGCACAATGTGAAG |
| *OjDFR1-32-F* | CGGAATTCATGGGAGTGGAGGATGCA |
| *OjDFR1-32-R* | CCCAAGCTTCTATTTTTGCTGGGATTC |
| *OjDFR1-121-F* | GCTCTAGAATGGGAGTGGAGGATGCA |
| *OjDFR1-121-R* | CGGGATCCCTATTTTTGCTGGGATTC |

* Restriction enzyme site are underlined

**Supplementary Table S2 HPLC-DAD and HPLC-ESI-MS analysis ofanthocyanin in acidic MeOH-H2O extracts of the wild-type *Arabidopsis***

| Peak number | Identifacation/tentative  identification | Retention time (min) | λmax (nm) | ESI-MS (m/z) | References |
| --- | --- | --- | --- | --- | --- |
| 1 | Cyanidin 3-*O*-[2*''*-*O*-(xylosyl) 6*''*-*O*-(*p*-*O*-(glucosyl) *p*-coumaroyl) glucoside] 5-*O*-[6*''''*-*O*-(malonyl) glucoside] | 48.78 | 266/531 | 287.1[Cy+H]^+^  1137.4[M+H]^+^ | Takayuki Tohge, Yasutaka Nishiyama et al., 2005 |
| 2 | Cyanidin 3-*O*-[2*''*-*O*-(6*'''*-*O*-(sinapoyl) xylosyl) 6*''*-*O*-(p-*O*-(glucosyl)-*p*-coumaroyl) glucoside] 5-*O*-(6*''''*-*O*-malonyl) glucoside | 50.84 | 269/535 | 287.0[Cy+H]^+^  1343.2[M+H]^+^ | Stephen J. Bloora, Sharon Abrahamsb., 2002 |
| 3 | Pelargonidin derivatives | 58.81 | 278/526 | 271.2[Pg+H]^+^ | Wei Sun, Xiangyu Meng et al., 2016 |
| 4 | Pelargonidin derivatives | 59.92 | 284/535 | 271.3[Pg+H]^+^ | Wei Sun, Xiangyu Meng et al., 2016 |
|  |  |  |  |  |  |
